# Prevalence of Self‐Reported Self‐Harm and Suicidal Ideation by Levels of Pain Intensity: An Analysis of the Australian National Survey of Mental Health and Wellbeing, 2020–2022

**DOI:** 10.1002/ejp.70449

**Published:** 2026-11-06

**Authors:** Wai Shun Fok, Scott Tagliaferri, Lianne Schmaal, Shu Mei Teo, Caroline X. Gao, Vivienne Browne, David G. Baker, Katrina Witt

**Affiliations:** ^1^ School of Population and Global Health The University of Melbourne Victoria Australia; ^2^ Centre for Youth Mental Health The University of Melbourne Victoria Australia; ^3^ Orygen The National Centre of Excellence in Youth Mental Health Victoria Australia; ^4^ Department of Epidemiology and Preventive Medicine, School of Public Health and Preventive Medicine Monash University Melbourne Victoria Australia

**Keywords:** Pain, pain management, pain medicine, self‐harm, suicidal ideation, suicide

## Abstract

**Background:**

Pain is common, and may co‐occur with suicide attempts, self‐harm and suicidal ideation. However, data on the prevalence of these outcomes across pain intensity levels is limited.

**Methods:**

Cross‐sectional analysis of the 2020–2022 National Survey of Mental Health and Wellbeing. Australian residents aged 16 to 85 years were recruited over two waves: December 2020 to July 2021 (*n* = 5554) and December 2021 to October 2022 (*n =* 10,339). We estimated the prevalence of self‐reported self‐harm or suicidal ideation in the past year by levels of recent pain intensity (none, mild, moderate, severe) in the past four weeks. Subgroup analyses were stratified by sex assigned at birth, age, presence of mental health conditions, linguistic diversity and geographic location.

**Results:**

Of the 15,880 (weighted) respondents with available data, 35% reported no pain, 41% mild pain, and 23% moderate or severe pain in the past four weeks. Past year self‐harm and suicidal ideation were reported by 2% and 3%, respectively. The prevalence of self‐harm increased with pain intensity: 0.9% (no pain), 1.9% (mild), 2.7% (moderate) and 3.5% (severe). The prevalence of suicidal ideation also increased with pain intensity: 1.6% (none), 3.0% (mild), 4.7% (moderate), 8.7% (severe). These patterns were largely consistent by sex and age group.

**Conclusions:**

Greater recent pain intensity is associated with higher prevalence of self‐reported self‐harm and suicidal ideation. Monitoring mental health among people experiencing pain is important, and integrating routine suicide risk assessment into pain management and physical health care settings could support targeted suicide prevention efforts and inform policy and clinical practice.

**Significance Statement:**

In this nationally representative Australian general population sample, we found a graded association between recent bodily pain and suicidal ideation and self‐harm. The consistency of this graded association across major demographic subgroups strengthens the case for pain intensity as a practical, low‐burden screening cue in primary care, pain management and physiotherapy settings. Enhanced training for general practitioners and physiotherapists should be a cornerstone of suicide prevention strategies.

## 1 Introduction

Pain is a leading contributor to global disease burden, with certain chronic pain conditions (e.g., lower back pain, headache disorders) among the leading causes of years lived with disability globally (GBD 2021 Diseases and Injuries Collaborators 2024). Worldwide, an estimated 10%–15% of people (random‐effects pooled point estimate: 10.6%) experience chronic pain (Mansfield et al. 2016), defined as persistent or recurrent pain lasting longer than three months (Treede et al. 2015), and an estimated 15.4% of Australians live with chronic pain (Deloitte Access Economics 2019), a figure projected to increase to 16.9% (5.23 million Australians) by 2050 (Deloitte Access Economics 2019). A larger proportion of adults in the general population report experiencing recent pain, defined as pain within the past 30 days, with estimates between 10%–50% (random‐effects pooled point estimate: 27.5%) (Zimmer et al. 2022). The associated social and health care costs of pain, estimated at $139.3 billion Australian dollars (AUD) in 2018 (both direct and indirect costs), are also projected to increase to $215.6 billion AUD by 2050 (Deloitte Access Economics 2019).

Both chronic and recent bodily pain have been associated with an increased odds of suicide, self‐harm and suicidal ideation. Globally, more than one million people die by suicide each year (Meda et al. 2025). In 2023, Australia reported 3307 suicide deaths (Australian Bureau of Statistics 2025b), giving an age‐standardised rate of 11.8 per 100,000 persons, higher than the most recent global average of 9.0 per 100,000 in 2021 (GBD 2021 Suicide Collaborators 2025). Both self‐harm and suicidal ideation are important risk factors for suicide. While some international studies report that greater pain intensity is associated with greater odds of reporting suicidal ideation (de Heer et al. 2020; Shen et al. 2024; Smith, Perlis, et al. 2004) and/or self‐harm (Ashrafioun et al. 2019; Grocott et al. 2021), even following adjustment for the presence of mental health conditions, others find no such association in chronic pain populations (Cho et al. 2013; Smith, Edwards, et al. 2004). Notably, there is limited data from nationally representative Australian general population samples estimating this association. Despite this gap in the evidence, a strong relationship between physical illness and suicide has been identified in the Australian National Suicide Prevention Strategy 2025–2035. Understanding the prevalence of self‐harm and suicidal ideation across levels of pain intensity could help guide services supporting people living with pain and highlight opportunities to integrate suicide risk screening and management into pain management and physical health care settings.

Multiple factors may influence the occurrence of self‐harm and/or suicidal ideation among people living with pain. According to the Interpersonal Theory of Suicide (Van Orden et al. 2010), pain‐related functional limitation and/or disability may heighten perceived burdensomeness and/or thwarted belongingness (Hirsch et al. 2016), both proximal drivers of the transition between suicidal ideation and action. Certain clinical, demographic and social factors have also been associated with the likelihood of experiencing suicidal ideation and/or self‐harm after pain. Males (Kwon and Lee 2023), those from Caucasian and/or Asian ethnic backgrounds (Kwon and Lee 2023), and those diagnosed with certain mental disorders, particularly anxiety, depressive or psychosis‐related disorders, may report a higher prevalence of self‐harm and/or suicidal ideation (Forget et al. 2026; Kwon and Lee 2023), whilst older adults and those from African and/or Hispanic/Latino/Latina ethnic backgrounds may report a lower prevalence (Kwon and Lee 2023). However, evidence for these modifiers derives predominately from clinical pain samples, rather than from community‐dwelling samples, and it therefore remains unclear whether similar patterns hold among those reporting pain in the general population. Rurality has also been independently associated with both greater pain burden (Mesa‐Castrillon et al. 2024) and greater suicide risk (Barry et al. 2020), but whether this factor influences the risk of suicidal ideation and/or non‐fatal suicidal behaviour among those experiencing pain has not been directly tested.

Additionally, the prevalence of self‐harm and suicidal ideation across levels of pain intensity is not well described, and comparatively few studies have evaluated whether prevalence follows a dose–response gradient across levels of pain intensity (Kwon and Lee 2023). Estimating these patterns could help guide healthcare services by identifying those who may benefit from targeted support and inform the design and allocation of appropriate resources to reduce healthcare inequalities (Karran et al. 2025). Therefore, our primary aim was to describe the prevalence of self‐harm and suicidal ideation across levels of recent pain intensity in a nationally representative Australian sample (*descriptive question*). Our secondary aim was to examine whether these prevalence patterns differ across key demographic subgroups, including sex assigned at birth, age, presence of mental health disorders, linguistic diversity and/or geographic location (*descriptive question*).

## 2 Methods

### 2.1 Study Design

This was a cross‐sectional analysis of data from the 2020–22 National Survey of Mental Health and Wellbeing (NSMHW) (Australian Bureau of Statistics 2024). The NSMHW, conducted by the Australian Bureau of Statistics (ABS), surveyed Australian residents on two occasions: December 2020 to July 2021 (Cohort 1), and December 2021 to October 2022 (Cohort 2). Data from these two cohorts were combined. Respondents provided verbal informed consent at the time of participation. As this study used pre‐collected data from the ABS, a retrospective waiver of consent was granted as obtaining consent from respondents a second time would have been impractical. Ethical approval was obtained via amendment to an existing project approved by The University of Melbourne's Human Research Ethics Committee on 26 March 2025 (2025‐25347‐64291‐7). This study is reported in accordance with the Strengthening the Reporting of Observational studies in Epidemiology (STROBE) guidelines (von Elm et al. 2008). See Supplementary Table 1.

### 2.2 Setting, Participants and Study Size

All usual residents of Australia aged 16–85 years living in private dwellings, in both urban and rural areas across all states and territories, were eligible for inclusion (Australian Bureau of Statistics 2024). Those living in very remote locations and discrete Aboriginal and Torres Strait Islander communities were excluded, as were temporary overseas visitors, members of non‐Australian defence forces and their households, non‐Australian diplomats and members of their households and those living in non‐private dwellings (e.g., custodial institutions). From each selected dwelling, one person aged 16–85 was randomly chosen to complete the survey. To improve estimate accuracy for individuals aged 16–24 years, this age group was given a higher probability of selection.

### 2.3 Data Sources, Measures and Variables

The NSMHW explored a range of areas, including self‐reported experiences of bodily pain, lifetime, 12‐month, and 30‐day prevalence of selected mental disorders. These were based on International Classification of Diseases, 10th Revision (ICD‐10) criteria (World Health Organization 2019) and the World Health Organisation Composite International Diagnostic Interview, version 3.0 (CIDI 3.0) (Kessler and Ustün 2004). Further items assessed lifetime and 12‐month prevalence of self‐harm and suicidal ideation. The survey also collected key demographic and socio‐economic characteristics, such as sex assigned at birth, age, main language spoken at home, and geographic location. Original survey items and response options are provided in Supplementary Table 2. Data were predominately collected through face‐to‐face interviews by trained ABS interviewers, although for 4.3% of households in Cohort 2, data collection was carried out by telephone due to pandemic stay‐at‐home orders (Australian Bureau of Statistics 2024).

### 2.4 Self‐Harm and Suicidal Ideation

*Self‐harm*: Given that non‐suicidal self‐injury and attempted suicide cannot be distinguished in any reliable way (Klonsky 2011), including on risk of suicide death (Waern et al. 2022) and that self‐reported motivations behind self‐harm change even within a single episode (De Beurs et al. 2018), consistent with international definitions we defined self‐harm as any deliberately self‐inflicted act of self‐poisoning and/or self‐injury whether or not there is evidence of suicidal intent (Hawton et al. 2003). This variable was formed by combining data from two items in the NSMHW: ‘Have you intentionally harmed yourself in any way, but not with the intention of taking your life in the last 12 months?’ and ‘Did you try to take your life in the last 12 months?’ Respondents could answer with: ‘not applicable’, ‘yes’, ‘no’, ‘not known’, or ‘refused’. If a respondent answered ‘yes’ to either question, they were coded as ‘yes’ for this outcome. If they answered ‘no’ to both questions, they were coded as ‘no’. Responses of ‘not applicable’, ‘not known’ and ‘refused’ were all recoded as missing.

*Suicidal ideation*: We defined suicidal ideation as thinking of suicide with or without suicidal intent, or hope for death by killing oneself without engaging in suicidal behaviour, consistent with international definitions (De Leo et al. 2021). We combined data from two items in the NSMHW to form this variable: ‘Have you seriously thought of taking your life in the last 12 months?*’* and ‘Did you make a plan about how you would end your life in the last 12 months?’ As with the self‐harm variable, respondents could answer with: ‘not applicable’, ‘yes’, ‘no’, ‘not known’, or ‘refused’. These response options were recoded to yes, no, or not applicable.

### 2.5 Bodily Pain Intensity

Bodily pain was assessed via responses to the following question: ‘Have you experienced bodily pain in the last four weeks?’. To simplify analysis and ensure compliance with ABS data suppression rules, responses were consolidated into four categories: ‘none’, ‘mild’ (i.e., combining ‘very mild’ and ‘mild’ response options), ‘moderate’ and ‘severe’ (i.e., combining ‘severe’ and ‘very severe’ response options). All other responses were recoded as missing.

### 2.6 Subgroup Variables

Five key demographic variables were selected for subgroup analysis based on evidence from the literature demonstrating their potential influence on both experiences of chronic pain and self‐harm and/or suicidal ideation, and their relevance for informing targeted screening and integration of suicide risk management within physical health services (Karran et al. 2025).

*Sex assigned at birth*: Biological sex was treated as a binary variable in this study. Due to the very small number of respondents identifying as non‐binary, these responses had to be excluded consistent with the data suppression rules imposed by the ABS. We report biological sex rather than gender, consistent with the predominant approach in the pain literature where sex‐based analyses are more commonly used to examine variation in pain‐related outcomes and associated risk factors (Kocot‐Kępska 2024).

*Age*: To ensure compliance with the ABS data suppression rules, age was transformed from a continuous variable into four categories, consistent with ABS definitions as: young people (i.e., those aged 16 to 24 years), lower middle adults (i.e., those aged 25 to 44 years), upper middle adults (i.e., those aged 45 to 64 years) and older adults (i.e., those aged 65 years and older).

*Presence of any mental health disorder*: Respondents were asked a series of questions about experiences of any mental health disorder in the past 30 days. The 30‐day timeframe was chosen to align closely with the four‐week period used for our exposure variable, pain intensity. Separate questions assessed the presence of any anxiety disorder, any affective disorder, or any substance use disorder specifically. Respondents reporting any mental health disorder were coded as ‘yes,’ whereas those reporting none were coded as ‘no.’

*Linguistic diversity*: We used respondents' main language spoken at home to identify linguistically diverse groups, consistent with the standard adopted by the ABS (Australian Bureau of Statistics 2018). Specifically, those respondents indicating that the main language spoken at home was a language other than English were classified as ‘linguistically diverse’, whilst those who spoke only English at home were classified as ‘non‐linguistically diverse’.

*Geographic location*. The Australian Statistical Geography Standard Remoteness Structure (ASGS) defines five classes of relative geographic remoteness across Australia (Australian Bureau of Statistics 2025a): ‘major cities’, ‘inner regional’, ‘outer regional’, ‘remote’ and ‘very remote’. In this study, remoteness was classified according to the ASGS structure but was recoded into a binary variable, ‘major cities’ versus ‘others’ to ensure compliance with ABS data suppression rules.

### 2.7 Statistical Methods

All analyses were conducted in the ABS secure DataLab environment using R for Windows, version 4.4.1. Population weights were applied to ensure that results accurately reflect the demographic and population profile of Australia. Descriptive analyses summarised absolute counts and percentages across four levels of bodily pain intensity (i.e., none, mild, moderate, severe). Prevalence of self‐reported self‐harm and suicidal ideation in the past year was calculated for each pain intensity group. Subgroup analyses were performed to examine descriptive patterns across sex assigned at birth, age, presence of any mental health disorders, linguistic diversity and geographic remoteness. In accordance with ABS data suppression rules, all counts were rounded to the nearest ten to prevent inadvertent re‐identification of respondents. Consistent with methodological guidance under which univariable regression models are an appropriate tool for quantifying the strength and precision of the patterns observed across pain intensity levels (Carlin and Moreno‐Betancur 2025), we report odds ratios (ORs) with 95% confidence intervals (CIs).

## 3 Results

### 3.1 Participants

Overall, 30,586 persons were approached to participate in the NSMHW. Cohort one comprised 5554 fully responding persons (57% response rate), and cohort two comprised 10,339 fully responding persons (50% response rate). Combined, this resulted in 15,893 persons overall, giving a total response rate of 52%. Of the 15,880 (weighted) persons with data available for analysis, half (51%, weighted) were female and two‐thirds (67%, weighted) were either younger adults (i.e., aged 25 to 44 years) or middle‐aged adults (i.e., aged 45 to 64 years). The majority (88%, weighted) reported no mental health diagnoses in the past 30 days. Most reported speaking only English at home (85%, weighted) whilst three‐quarters (76%, weighted) resided in major cities (Table 1).

**TABLE 1:** Characteristics of respondents by bodily pain intensity in the last four weeks.

Characteristics	Bodily pain in the last four weeks				Overall	*p*‐value^b^
	None	Mild	Moderate	Severe		
	*N* = 5,540^a^	*N* = 6,640^a^	*N* = 2,560^a^	*N* = 1,150^a^	*N* = 15,880^a^	
Sex assigned at birth						< 0.001
Male	2920 (53%)	3350 (51%)	1080 (42%)	460 (40%)	7820 (49%)	
Female	2620 (47%)	3280 (49%)	1480 (58%)	680 (60%)	8060 (51%)	
Age group						< 0.001
Young people	1090 (20%)	820 (12%)	190 (7%)	70 (6%)	2170 (14%)	
Lower middle adult	2250 (41%)	2420 (36%)	760 (30%)	290 (25%)	5720 (36%)	
Upper middle adult	1430 (26%)	2180 (33%)	900 (35%)	450 (39%)	4950 (31%)	
Older adult	780 (14%)	1210 (18%)	710 (28%)	350 (30%)	3040 (19%)	
Mental health conditions, last 30 days						< 0.001
No	5130 (93%)	5880 (89%)	2110 (83%)	840 (73%)	13,960 (88%)	
Yes	410 (7%)	760 (11%)	440 (17%)	310 (27%)	1920 (12%)	
Main language spoken at home						< 0.001
English	4430 (80%)	5740 (87%)	2280 (89%)	1040 (91%)	13,490 (85%)	
Others	1110 (20%)	890 (13%)	280 (11%)	100 (9%)	2390 (15%)	
Geographical location						0.03
Major cities	4140 (75%)	5050 (76%)	2010 (79%)	890 (77%)	12,090 (76%)	
Others	1400 (25%)	1580 (24%)	550 (21%)	260 (23%)	3790 (24%)	
Self‐harm, last 12 months						< 0.001
No	5480 (99%)	6490 (98%)	2480 (97%)	1100 (97%)	15,550 (98%)	
Yes	50 (1%)	130 (2%)	70 (3%)	40 (3%)	300 (2%)	
Suicidal ideation, last 12 months						< 0.001
No	5430 (98%)	6410 (97%)	2430 (95%)	1040 (91%)	15,320 (97%)	
Yes	90 (2%)	200 (3%)	120 (5%)	100 (9%)	510 (3%)	

*Note:* All numbers are weighted and rounded to the nearest 10.

^a^ *n* (%).

^b^ Pearson's X^2: Rao & Scott adjustment; Design‐based Kruskal‐Wallis test.

Just over one‐third (35%, weighted) reported experiencing no bodily pain symptoms in the past four weeks, 41% (weighted) reported experiencing only mild pain over this same period, whilst just under one‐quarter (23%, weighted) reported experiencing either moderate or severe bodily pain over the past four weeks (Table 1; Supplementary Table 3). In the past 12 months, only a small proportion of respondents reported either self‐harm (2%, weighted) or suicidal ideation (3%, weighted) (Table 1; Supplementary Table 4).

### 3.2 Prevalence of Self‐Harm by Bodily Pain Intensity

Self‐reported prevalence of self‐harm in the past 12 months increased with higher pain intensity. Among respondents reporting no pain over the past four weeks, 0.9% reported self‐harm, compared with 1.9% of those reporting mild pain, 2.7% of those reporting moderate pain, and 3.5% of those reporting severe pain. In absolute terms, those reporting severe pain were roughly four times as likely to report self‐harm as compared to those reporting no recent bodily pain (Table 1; Figure 1).

**FIGURE 1:**
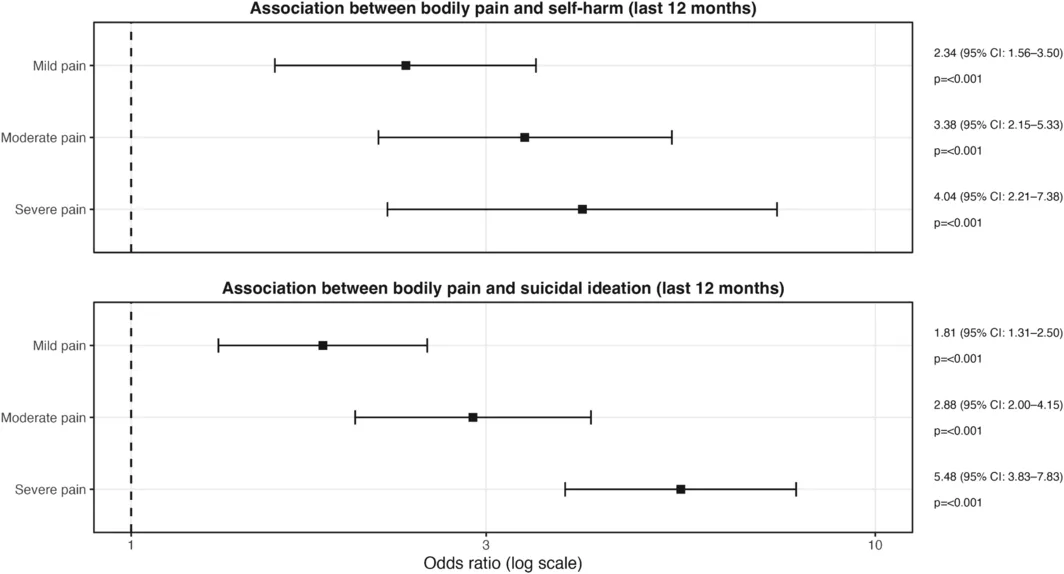
Odds ratios (OR) and 95% confidence intervals (CI) from univariable logistic regression models estimating the prevalence of self‐reported self‐harm and suicidal ideation by pain intensity.

### 3.3 Prevalence of Suicidal Ideation by Bodily Pain Intensity

A similar pattern was observed for suicidal ideation. Only 1.6% of respondents reporting no pain over the past four weeks reported suicidal ideation, compared with 3.0% for those reporting mild pain, 4.7% for those reporting moderate pain, and 8.7% for those reporting severe pain, meaning that the odds of reporting suicidal ideation were over five times higher among those reporting severe pain compared with those reporting no recent bodily pain (Table 1; Figure 1).

### 3.4 Subgroup Analyses

These patterns were generally consistent across subgroups defined by sex assigned at birth, age, presence of mental health conditions, linguistic diversity and geographic remoteness (Supplementary Tables 5–10).

## 4 Discussion

In this nationally representative cohort of 15,880 community‐dwelling Australian residents aged 16 to 85 years, we found that greater pain intensity over the past four weeks was associated with a higher prevalence of both self‐harm and suicidal ideation over the past 12 months. While prior research has focused on links between chronic pain and suicidal outcomes, comparatively fewer have evaluated differences in prevalence across levels of pain intensity (Kwon and Lee 2023).

We also examined whether these broad patterns varied by age group, sex, mental health status, linguistic diversity and geography because these characteristics are recommended equity‐relevant variables for pain research (Karran et al. 2025). Overall, prevalence patterns across these subgroups were broadly consistent. For example, although females reported higher prevalence of moderate or severe pain and were also more likely than males to report self‐harm and suicidal ideation, the overall pattern of increasing prevalence with pain intensity was similar across sexes, consistent with prior research in those experiencing migraine pain (Kuan et al. 2025). Patterns were also largely consistent across age groups, consistent with previous work reporting that pain‐related suicidal ideation and suicide attempts are more strongly linked among older adults than younger populations (Torino et al. 2025). For individuals from linguistically diverse backgrounds and those living in regional or remote areas, prevalence estimates were less precise due to smaller numbers. Mental health comorbidity has been proposed to explain the association between pain, self‐harm and suicide, with some studies showing associations are largely mediated by mental illness, particularly major depression (Balit et al. 2024; Liu et al. 2026). Our findings are more nuanced. For self‐harm, the pain intensity gradient was evident regardless of mental health status whilst for suicidal ideation, the gradient was pronounced among respondents without a current mental health condition. This suggests current mental health status may modify, rather than uniformly account for, the relationship between pain intensity and suicide‐related outcomes.

The mechanisms underlying these patterns are likely multifactorial. Increasing pain intensity may contribute to greater functional limitation, potentially influencing feelings of perceived burdensomeness, thwarted belongingness and hopelessness; all of which are key constructs within the Interpersonal Theory of Suicidal Behaviour (Van Orden et al. 2010). Equally plausible, however, is a bidirectional relationship, whereby psychological distress and/or mental ill‐health heightens pain perception, symptom reporting, or care seeking for pain. Because our cross‐sectional design cannot determine which exposure came first, our findings should be interpreted as describing co‐occurrence, rather than a causal relationship. Importantly, our findings do suggest that those presenting to services with greater pain intensity may report higher prevalence of self‐harm and suicidal ideation, highlighting opportunities for targeted clinical support and screening within chronic pain and physical health care settings.

### 4.1 Strengths and Limitations

The NSMHW is a large and nationally representative sample of the Australian population. Furthermore, the use of a validated measure, such as the ICD‐10 CIDI 3.0, to assess for both lifetime and 30‐day mental health symptoms is a strength. Subgroup analyses provide descriptive insights across key demographics, which can inform service planning (Karran et al. 2025). However, our study did rely on self‐reported measures for bodily pain intensity, self‐harm and suicidal ideation. Recall bias could have affected reporting of these factors. Additionally, bodily pain was reported over the past four weeks, while self‐harm and suicidal ideation were reported over 12 months, creating a discrepancy in recall periods. Whilst it is also possible that attrition, non‐response, and/or social desirability bias could have affected the reporting of some outcomes from the NSMHW, including self‐harm and/or suicidal ideation and recent bodily pain, the overall prevalence of recent bodily pain, self‐harm and suicidal ideation reported in this study are consistent with estimates from other Australian general population samples (Laß et al. 2025; Pirkis et al. 2000).

Finally, some key variables, such as bodily location, pain type, and pain catastrophising were not recorded even though existing literature suggests these might influence the occurrence of self‐harm and/or suicidal ideation across different pain disorders (Adawi et al. 2021; Giakas et al. 2023; Kwon and Lee 2023; Themelis et al. 2023). Additionally, whilst our composite mental health variable included substance use disorder, we were unable capture information on opioid and/or other analgesic use disorder specifically. This is relevant as evidence suggests that opioid use disorder, but not opioid use per se, may be linked with elevated suicide risk in chronic pain populations (Cheatle et al. 2023). The absence of these variables may therefore limit the generalisability of our findings to specific clinical groups, such as those experiencing neuropathic pain or complex opioid‐related pain disorders, whose risk profiles may differ from general community samples.

### 4.2 Implications

The above findings have important implications for public health and suicide prevention policies and clinical practice. Routine enquiry about self‐harm and suicidal ideation among people experiencing pain, particularly those reporting higher pain intensity, could help identify individuals with elevated prevalence of these outcomes. Primary care providers, including general practitioners (GPs) and physiotherapists, are well‐placed to initiate these conversations due to their frequent and often ongoing contact with patients (Large and Myles 2017; Vancampfort et al. 2025). This is particularly important given emerging evidence that many people who self‐harm do not receive specialist mental health follow‐up. A recent Australian data‐linkage study found that around one‐quarter of those presenting to emergency departments after self‐harm only receive care from physical health providers in the 28 days following their presentation, rising to just over one‐third within one year (Witt et al. 2026).

Almost all (93.2%) GPs report encountering suicidal patients at least once over the course of their careers (Bajaj et al. 2008). Further, half (49.8%) of all physiotherapists, and almost all (93.8%) physiotherapists working in specialist pain management clinics, report treating clients who disclosed suicidal thoughts at least once over the course of their careers (McGrath et al. 2023). Despite this, many GPs and physiotherapists do not feel adequately equipped to screen for suicidal ideation and/or behaviours effectively. In part, this is because formal training for screening for suicidal ideation and behaviour is often lacking for these professional groups. For example, when asked if they received any training, many GPs (60.2%) and physiotherapists (55.9%) reported they have not received any formal training to identify those who might be experiencing suicidal ideation and/or behaviour (Bajaj et al. 2008; McGrath et al. 2023). Suicide prevention training is not consistently included in entry‐level physiotherapy curricula (Vancampfort et al. 2025). Similarly, among GPs who report receiving any such training, approximately half indicate it occurred during their specialist psychiatry placements, which may have taken place several years earlier (Bajaj et al. 2008).

Governments should allocate funding to incentivise GPs and physiotherapists to undertake Continuing Professional Development modules that address the relationship between comorbid physical pain, self‐harm and suicide‐related behaviours/risk to support identification and inform treatment approaches. Equipping GPs and physiotherapists with the skills and confidence to broach the subject of self‐harm and suicide‐related behaviours/risk with patients presenting with pain could be an effective suicide prevention strategy for this population group.

## 5 Conclusion

Higher self‐reported recent pain intensity was associated with higher prevalence of both self‐harm and suicidal ideation over the past 12 months. Prevalence patterns were generally consistent across subgroups, although estimates were less precise for older adults, those from linguistically diverse backgrounds, and those from regional or remote areas. Given that pain intensity is routinely assessed in primary care and physiotherapy consultations, using it to trigger suicide risk screening would not require adding a new instrument to clinical practice, yet could meaningfully extend the reach of suicide prevention efforts beyond mental health services. Realising this will require investment in training for GPs and physiotherapists, who are often the first point of contact for people in pain.

## Author Contributions

Conceptualisation: Scott Tagliaferri, Katrina Witt; Data curation: Not applicable; Formal analysis: Wai Shun Fok; Funding acquisition: Not applicable; Investigation: Wai Shun Fok, Scott Tagliaferri, Katrina Witt; Methodology: Scott Tagliaferri, Katrina Witt, Lianne Schmaal, Shu Mei Teo, Caroline X. Gao, Katrina Witt; Project administration: Not applicable; Resources: Scott Tagliaferri, Katrina Witt. Software: Scott Tagliaferri, Katrina Witt; Supervision: Scott Tagliaferri, Katrina Witt; Validation: Katrina Witt, Caroline X. Gao; visualisation: Wai Shun Fok; Writing – original draft: Wai Shun Fok, Scott Tagliaferri, Katrina Witt; Writing – review and editing: All. Final manuscript approved by all authors.

## Funding

Whilst no specific funding was received for this work, individual authors wish to acknowledge the following sources of support. SDT is supported by a Sir Randal Heymanson Fellowship from The University of Melbourne. KW is supported by the Dame Kate Campbell Fellowship from the Faculty of Medicine, Dentistry, and Health Sciences, The University of Melbourne. Orygen is funded by the Australian Government Department of Health, Disability and Ageing to provide technical advice and policy direction on youth mental health topics (VB and DGB).

## Conflicts of Interest

The authors declare no conflicts of interest.

## Supporting information


**Supplementary Table 1** Strengthening the reporting of observational studies in epidemiology (STROBE) checklist.
**Supplementary Table 2**. Survey items and response options from the National Survey of Mental Health and Wellbeing for all variables used in this study.
**Supplementary Table 3**. Characteristics of respondents reporting self‐harm in the last 12 months.
**Supplementary Table 4**. Characteristics of respondents reporting suicidal ideation in the last 12 months.
**Supplementary Table 5**. Sub‐group analyses by sex assigned at birth.
**Supplementary Table 6**. Sub‐group analyses by age group, self‐harm in the last 12 months.
**Supplementary Table 7**. Sub‐group analyses by age group, suicidal ideation in the last 12 months.
**Supplementary Table 8**. Sub‐group analyses by presence of any mental health conditions in the last thirty days.
**Supplementary Table 9**. Sub‐group analyses by main language spoken at home.
**Supplementary Table 10**. Sub‐group analyses by geographic location.

## Data Availability

Data from the National Study of Mental Health and Wellbeing (NSMHW) are available from the Australian Bureau of Statistics (ABS) upon request.
