# Supplementary material for: Prevalence of Self‐Reported Self‐Harm and Suicidal Ideation by Levels of Pain Intensity: An Analysis of the Australian National Survey of Mental Health and Wellbeing, 2020–2022
Source: Eur J Pain. 2026 Nov 6;30(10):e70449. doi: 10.1002/ejp.70449 (PMC13741988; doi:10.1002/ejp.70449)
Supplement: Supplementary file 1 [file EJP-30-0-s001.docx]

**Supplementary Table 1.** Strengthening the reporting of observational studies in epidemiology (STROBE) checklist.

|  | **Item No.** | **Recommendation** | **Page** |
| --- | --- | --- | --- |
| **Title and abstract** |  |  |  |
|  | 1 | (*a*) Indicate the study’s design with a commonly used term in the title or the abstract | 2 |
|  |  | (*b*) Provide in the abstract an informative and balanced summary of what was done and what was found | 2 |
| Background/rationale | 2 | Explain the scientific background and rationale for the investigation being reported | 3 |
| Objectives | 3 | State specific objectives, including any prespecified hypotheses | 4 |
| **Methods** |  |  |  |
| Study design | 4 | Present key elements of study design early in the paper | 4 |
| Setting | 5 | Describe the setting, locations, and relevant dates, including periods of recruitment, exposure, follow-up, and data collection | 5 |
| Participants | 6 | (*a*) *Cohort study*—Give the eligibility criteria, and the sources and methods of selection of participants. Describe methods of follow-up  *Case-control study*—Give the eligibility criteria, and the sources and methods of case ascertainment and control selection. Give the rationale for the choice of cases and controls  *Cross-sectional study*—Give the eligibility criteria, and the sources and methods of selection of participants | 5 |
|  |  | (*b*) *Cohort study*—For matched studies, give matching criteria and number of exposed and unexposed  *Case-control study*—For matched studies, give matching criteria and the number of controls per case | N/A |
| Variables | 7 | Clearly define all outcomes, exposures, predictors, potential confounders, and effect modifiers. Give diagnostic criteria, if applicable | 5-6 |
| Data sources/ measurement | 8* | For each variable of interest, give sources of data and details of methods of assessment (measurement). Describe comparability of assessment methods if there is more than one group | 5-7 |
| Bias | 9 | Describe any efforts to address potential sources of bias | 5-7 |
| Study size | 10 | Explain how the study size was arrived at | 8 |
| Quantitative variables | 11 | Explain how quantitative variables were handled in the analyses. If applicable, describe which groupings were chosen and why | 8 |
| Statistical methods | 12 | (*a*) Describe all statistical methods, including those used to control for confounding | 8 |
|  |  | (*b*) Describe any methods used to examine subgroups and interactions | 9 |
|  |  | (*c*) Explain how missing data were addressed | 8-9 |
|  |  | (*d*) *Cohort study*—If applicable, explain how loss to follow-up was addressed  *Case-control study*—If applicable, explain how matching of cases and controls was addressed  *Cross-sectional study*—If applicable, describe analytical methods taking account of sampling strategy | 8-9 |
|  |  | (*e*) Describe any sensitivity analyses |  |
| **Results** |  |  |  |
| Participants | 13* | (a) Report numbers of individuals at each stage of study—eg numbers potentially eligible, examined for eligibility, confirmed eligible, included in the study, completing follow-up, and analysed | 8 |
|  |  | (b) Give reasons for non-participation at each stage | 8 |
|  |  | (c) Consider use of a flow diagram | N/A |
| Descriptive data | 14* | (a) Give characteristics of study participants (eg demographic, clinical, social) and information on exposures and potential confounders | 8 |
|  |  | (b) Indicate number of participants with missing data for each variable of interest | Table 1 |
|  |  | (c) *Cohort study*—Summarise follow-up time (eg, average and total amount) | N/A |
| Outcome data | 15* | *Cohort study*—Report numbers of outcome events or summary measures over time | 9 |
|  |  | *Case-control study—*Report numbers in each exposure category, or summary measures of exposure | N/A |
|  |  | *Cross-sectional study—*Report numbers of outcome events or summary measures | Table 1 |
| Main results | 16 | (*a*) Give unadjusted estimates and, if applicable, confounder-adjusted estimates and their precision (eg, 95% confidence interval). Make clear which confounders were adjusted for and why they were included | Table 1 |
|  |  | (*b*) Report category boundaries when continuous variables were categorised | N/A |
|  |  | (*c*) If relevant, consider translating estimates of relative risk into absolute risk for a meaningful time period | N/A |
| Other analyses | 17 | Report other analyses done—eg analyses of subgroups and interactions, and sensitivity analyses | Supplementary Tables |
| **Discussion** | | | |
| Key results | 18 | Summarise key results with reference to study objectives | 10 |
| Limitations | 19 | Discuss limitations of the study, taking into account sources of potential bias or imprecision. Discuss both direction and magnitude of any potential bias | 10 |
| Interpretation | 20 | Give a cautious overall interpretation of results considering objectives, limitations, multiplicity of analyses, results from similar studies, and other relevant evidence | 10 |
| Generalisability | 21 | Discuss the generalisability (external validity) of the study results | 10 |
| **Other information** |  | | |
| Funding | 22 | Give the source of funding and the role of the funders for the present study and, if applicable, for the original study on which the present article is based | 1 |

**Supplementary Table 2.** Survey items and response options from the National Survey of Mental Health and Wellbeing for all variables used in this study.

|  | **Item Label and Response Options** | **Population** |
| --- | --- | --- |
| **THSUIC12** |  |  |
|  | Whether seriously thought about taking own life in last 12 months | Persons who had seriously thought about taking their own life |
|  | 0. Not applicable |  |
|  | 1. Yes |  |
|  | 5. No |  |
|  | 8. Not known |  |
|  | 9. Refused |  |
| **PLSUIC12** |  |  |
|  | Whether made a plan for taking own life in last 12 months | Persons who had made a plan for taking their own life |
|  | 0. Not applicable |  |
|  | 1. Yes |  |
|  | 5. No |  |
|  | 8. Not known |  |
|  | 9. Refused |  |
| **SH12MNTH** |  |  |
|  | Whether self-harmed in last 12 months | Persons who had ever self-harmed |
|  | 0. Not applicable |  |
|  | 1. Yes |  |
|  | 5. No |  |
|  | 8. Not known |  |
|  | 9. Refused |  |
| **ATSUIC12** |  |  |
|  | Whether attempted to take own life in last 12 months | Persons who had attempted to take their own life |
|  | 0. Not applicable |  |
|  | 1. Yes |  |
|  | 5. No |  |
|  | 8. Not known |  |
|  | 9. Refused |  |
| **PAINQ01** |  |  |
|  | Bodily pain experienced in the last four weeks | All persons |
|  | 1. None |  |
|  | 2. Very mild |  |
|  | 3. Mild |  |
|  | 4. Moderate |  |
|  | 5. Severe |  |
|  | 6. Very severe |  |
|  | 8. Not known |  |
|  | 9. Refused |  |
| **AGEP** |  |  |
|  | Age of person | All persons |
|  | Age in single years until values <0….98> |  |
|  | 99 years and over |  |
| **SEXSPS** |  |  |
|  | Sex at birth | All persons |
|  | 1.Male |  |
|  | 2.Female |  |
|  | 3.Another term |  |
| **I_COH30** |  |  |
|  | Comorbidity of ICD-10 Disorder Classes in the past 30 days (with hierarchy) | All persons |
|  | 1.Affective disorder only |  |
|  | 2.Anxiety disorder only |  |
|  | 3.Substance use disorder only |  |
|  | 4.Affective and anxiety disorders only |  |
|  | 5.Affective and substance use disorders only |  |
|  | 6.Anxiety and substance use disorders only |  |
|  | 7.Affective, anxiety, and substance use only |  |
|  | 8.No mental disorder |  |
| **MAILANGU** |  |  |
|  | Main language spoken at home | All persons |
|  | 10.English |  |
|  | 11.Mandarin |  |
|  | 12.Arabic |  |
|  | 13.Cantonese |  |
|  | 14.Vietnamese |  |
|  | 15.Italian |  |
|  | 16.Greek |  |
|  | 17.Hindi |  |
|  | 18.Spanish |  |
|  | 19.Punjabi |  |
|  | 22.Other |  |
| **ARIA** |  |  |
|  | Remoteness area (ARIA) – ASGS 2016 | All persons |
|  | 0.Major cities of Australia |  |
|  | 1.Inner regional Australia |  |
|  | 2.Outer regional Australia |  |
|  | 3.Remote Australia |  |
|  | 4.Very remote Australia |  |
|  | 5.Migratry – Offshore - Shipping |  |
|  | 9.No usual address |  |

**Supplementary Table 3.** Characteristics of respondents reporting self-harm in the last 12 months.

| **Characteristics** | **Self-harm and/or suicide attempt in the last 12 months** | | | |
| --- | --- | --- | --- | --- |
|  | **No** | **Yes** | **Overall** | **P-value^2^** |
|  | **N=15,560^1^** | **N=300^1^** | **N=15,850^1^** |  |
| **Sex assigned at birth** |  |  |  | 0.003 |
| Male | 7,710 (50%) | 110 (38%) | 7,820 (49%) |  |
| Female | 7,850 (50%) | 180 (62%) | 8,030 (51%) |  |
| **Age** |  |  |  | <0.001 |
| Young people | 2,020 (13%) | 140 (48%) | 2,170 (14%) |  |
| Lower middle adult | 5,590 (36%) | 110 (38%) | 5,710 (36%) |  |
| Upper middle adult | 4,900 (31%) | 40 (26%) | 4,940 (31%) |  |
| Older adult | 3,030 (20%) | 0 (2%) | 3,040 (19%) |  |
| **Bodily pain in the last four weeks** |  |  |  | <0.001 |
| None | 5,480 (35%) | 50 (16%) | 5,520 (35%) |  |
| Mild | 6,490 (42%) | 130 (45%) | 6,620 (42%) |  |
| Moderate | 2,480 (16%) | 70 (25%) | 2,560 (16%) |  |
| Severe | 1,100 (7%) | 40 (13%) | 1,140 (7%) |  |
| **Mental health condition, last 30 days** |  |  |  | <0.001 |
| No | 13,830 (89%) | 100 (36%) | 13,930 (88%) |  |
| Yes | 1,730 (11%) | 190 (64%) | 1,920 (12%) |  |
| **Main language spoken at home** |  |  |  | <0.001 |
| English | 13,180 (85%) | 280 (96%) | 13,470 (85%) |  |
| Others | 2,370 (15%) | 10 (4%) | 2,390 (15%) |  |
| **Geographic location** |  |  |  | 0.104 |
| Major cities | 11,820 (76%) | 240 (81%) | 12,060 (76%) |  |
| Others | 3,730 (24%) | 60 (19%) | 3,790 (24%) |  |
| **Suicidal ideation, last 12 months** |  |  |  | <0.001 |
| No | 15,140 (97%) | 180 (60%) | 15,310 (97%) |  |
| Yes | 400 (3%) | 120 (40%) | 520 (3%) |  |

*Table notes:* All numbers are weighted and rounded to the nearest 10.

^1^ n (%).

^2^ Pearson’s X^2: Rao & Scott adjustment; Design-based Kruskal-Wallis test.

**Supplementary Table 4.** Characteristics of respondents reporting suicidal ideation in the last 12 months.

| **Characteristics** | **Suicidal ideation and/or plans in the last 12 months** | | | |
| --- | --- | --- | --- | --- |
|  | **No** | **Yes** | **Overall** | **P-value^2^** |
|  | **N=15,320^1^** | **N=520^1^** | **N=15,840^1^** |  |
| **Sex assigned at birth** |  |  |  | <0.211 |
| Male | 7,570 (49%) | 240(46%) | 7,810 (49%) |  |
| Female | 7,750 (51%) | 280 (54%) | 8,030 (51%) |  |
| **Age** |  |  |  | <0.001 |
| Young people | 2,030 (13%) | 140 (27%) | 2,170 (14%) |  |
| Lower middle adult | 5,530 (36%) | 170 (33%) | 5,700 (36%) |  |
| Upper middle adult | 4,800 (31%) | 140 (26%) | 4,940 (31%) |  |
| Older adult | 2,970 (19%) | 70 (13%) | 3,040 (19%) |  |
| **Bodily pain in the last four weeks** |  |  |  | <0.001 |
| None | 5,430 (35%) | 90 (18%) | 5,520 (35%) |  |
| Mild | 6,410 (42%) | 200 (39%) | 6,610 (42%) |  |
| Moderate | 2,430 (16%) | 120 (24%) | 2,560 (16%) |  |
| Severe | 1,040 (7%) | 100 (19%) | 1,140 (7%) |  |
| **Mental health condition, last 30 days** |  |  |  | <0.001 |
| No | 13,690 (89%) | 230 (45%) | 13,920 (88%) |  |
| Yes | 1,630 (11%) | 290 (55%) | 1,920 (12%) |  |
| **Main language spoken at home** |  |  |  | <0.001 |
| English | 12,970 (85%) | 500 (95%) | 13,460 (85%) |  |
| Others | 2,360 (15%) | 20 (5%) | 2,380 (15%) |  |
| **Geographic location** |  |  |  | 0.998 |
| Major cities | 11,660 (76%) | 400 (76%) | 12,050 (76%) |  |
| Others | 3,670 (24%) | 120 (24%) | 3,790 (24%) |  |
| **Suicidal ideation, last 12 months** |  |  |  | <0.001 |
| No | 15,140 (99%) | 400 (77%) | 15,540 (98%) |  |
| Yes | 180 (1%) | 120 (23%) | 300 (2%) |  |

*Table notes:* All numbers are weighted and rounded to the nearest 10.

^1^ n (%).

^2^ Pearson’s X^2: Rao & Scott adjustment; Design-based Kruskal-Wallis test.

**Supplementary Table 5.** Sub-group analyses by sex assigned at birth.

|  | **Self-harm in the last twelve months** | | | | **Suicidal ideation in the last twelve months** | | | |
| --- | --- | --- | --- | --- | --- | --- | --- | --- |
| **Sex assigned at birth** | **Male** | | **Female** | | **Male** | | **Female** | |
| **Bodily pain in the last 4 weeks** | **Odds ratio** | **95% CI** | **Odds ratio** | **95% CI** | **Odds ratio** | **95% CI** | **Odds ratio** | **95% CI** |
| None (Reference) | - | - | - | - | - | - | - | - |
| Mild pain | 2.27 | 1.17 - 4.42 | 2.33 | 1.40 - 3.89 | 1.73 | 1.07 - 2.80 | 1.88 | 1.23 - 2.88 |
| Moderate pain | 4.82 | 2.40 - 9.68 | 2.52 | 1.33 - 4.61 | 3.11 | 1.72 - 5.60 | 2.69 | 1.71 - 4.23 |
| Severe pain | 4.00 | 1.14 - 14.02 | 3.70 | 1.87 - 7.32 | 5.02 | 2.83 - 8.89 | 5.72 | 3.62 - 9.04 |
| R^2^/R^2^ adjusted | 0.029/0.029 | | 0.017/0.017 | | 0.028/0.028 | | 0.033/0.033 | |

**Supplementary Table 6.** Sub-group analyses by age group, self-harm in the last 12 months.

|  | **Self-harm in the last twelve months** | | | | | | | |
| --- | --- | --- | --- | --- | --- | --- | --- | --- |
| **Age group** | **Young people** | | **Lower middle adult** | | **Upper middle adult** | | **Older adult** | |
| **Bodily pain in the last 4 weeks** | **Odds ratio** | **95% CI** | **Odds ratio** | **95% CI** | **Odds ratio** | **95% CI** | **Odds ratio** | **95% CI** |
| None | - | - | - | - | - | - | - | - |
| Mild pain | 2.50 | 1.45 - 4.32 | 5.34 | 2.73 - 10.40 | 4.01 | 1.17 - 13.70 | 0.54 | 0.05 - 5.40 |
| Moderate pain | 5.29 | 2.69 - 10.39 | 10.82 | 5.16 - 22.71 | 8.00 | 2.53 - 25.31 | na | na |
| Severe pain | 6.60 | 2.31 - 18.86 | 8.68 | 3.22 - 23.41 | 19.67 | 5.06 - 76.50 | 7.56 | 0.78 - 73.16 |
| R^2^/R^2^ adjusted | 0.051/0.049 | | 0.058/0.058 | | 0.063/0.062 | | 0.124/0.123 | |

|  | **Self-harm in the last twelve months** | | | | **Suicidal ideation in the last twelve months** | | | |
| --- | --- | --- | --- | --- | --- | --- | --- | --- |
| **Sex assigned at birth** | **Male** | | **Female** | | **Male** | | **Female** | |
| **Bodily pain in the last 4 weeks** | **Odds ratio** | **95% CI** | **Odds ratio** | **95% CI** | **Odds ratio** | **95% CI** | **Odds ratio** | **95% CI** |
| None (Reference) | - | - | - | - | - | - | - | - |
| Mild pain | 2.27 | 1.17 - 4.42 | 2.33 | 1.40 - 3.89 | 1.73 | 1.07 - 2.80 | 1.88 | 1.23 - 2.88 |
| Moderate pain | 4.82 | 2.40 - 9.68 | 2.52 | 1.33 - 4.61 | 3.11 | 1.72 - 5.60 | 2.69 | 1.71 - 4.23 |
| Severe pain | 4.00 | 1.14 - 14.02 | 3.70 | 1.87 - 7.32 | 5.02 | 2.83 - 8.89 | 5.72 | 3.62 - 9.04 |
| R^2^/R^2^ adjusted | 0.029/0.029 | | 0.017/0.017 | | 0.028/0.028 | | 0.033/0.033 | |

**Supplementary Table 7.** Sub-group analyses by age group, suicidal ideation in the last 12 months.

|  | **Suicidal ideation in the last twelve months** | | | | | | | |
| --- | --- | --- | --- | --- | --- | --- | --- | --- |
| **Age** | **Young people** | | **Lower middle adult** | | **Upper middle adult** | | **Older adult** | |
| **Bodily pain in the last 4 weeks** | **Odds ratio** | **95% CI** | **Odds ratio** | **95% CI** | **Odds ratio** | **95% CI** | **Odds ratio** | **95% CI** |
| None (Reference) | - | - | - | - | - | - | - | - |
| Mild pain | 2.11 | 1.20 - 3.69 | 1.66 | 1.01 - 2.71 | 2.83 | 1.28 - 6.25 | 3.51 | 1.35 - 9.14 |
| Moderate pain | 4.09 | 2.05 - 8.18 | 3.59 | 2.06 - 6.24 | 4.56 | 1.80 - 11.59 | 5.08 | 2.01 - 12.88 |
| Severe pain | 3.97 | 1.40 - 11.29 | 7.21 | 3.99 - 13.00 | 13.15 | 6.19 - 27.91 | 8.89 | 3.31 - 23.85 |
| R^2^/R^2^ adjusted | 0.033/0.032 | | 0.043/0.043 | | 0.064/0.064 | | 0.038/0.037 | |

**Supplementary Table 8.** Sub-group analyses by presence of any mental health conditions in the last thirty days.

| **Any mental health condition, last 30 days** | **Self-harm in the last twelve months** | | | | **Suicidal ideation in the last twelve months** | | | |
| --- | --- | --- | --- | --- | --- | --- | --- | --- |
|  | **No** | | **Yes** | | **No** | | **Yes** | |
| **Bodily pain in the last 4 weeks** | **Odds ratio** | **95% CI** | **Odds ratio** | **95% CI** | **Odds ratio** | **95% CI** | **Odds ratio** | **95% CI** |
| None (Reference) | - | - | - | - | - | - | - | - |
| Mild pain | 2.33 | 1.40 - 3.89 | 2.27 | 1.17 - 4.42 | 2.20 | 1.40 - 3.46 | 1.02 | 0.64 - 1.64 |
| Moderate pain | 2.52 | 1.33 - 4.61 | 4.82 | 2.40 - 9.68 | 2.86 | 1.75 - 4.68 | 1.35 | 0.79 - 2.31 |
| Severe pain | 3.70 | 1.87 - 7.32 | 4.00 | 1.14 - 14.02 | 6.85 | 4.15 - 11.32 | 1.49 | 0.88 - 2.53 |
| R^2^/R^2^ adjusted | 0.017/0.017 | | 0.029/0.029 | | 0.031/0.031 | | 0.004/0.002 | |

|  | **Self-harm in the last twelve months** | | | | **Suicidal ideation in the last twelve months** | | | |
| --- | --- | --- | --- | --- | --- | --- | --- | --- |
| **Main language spoken at home** | **English** | | **Others** | | **English** | | **Others** | |
| **Bodily pain in the last 4 weeks** | **Odds ratio** | **95% CI** | **Odds ratio** | **95% CI** | **Odds ratio** | **95% CI** | **Odds ratio** | **95% CI** |
| None (Reference) | - | - | - | - | - | - | - | - |
| Mild pain | 2.12 | 1.40 - 3.21 | 5.74 | 1.13 - 29.11 | 1.78 | 1.27 - 2.48 | 1.23 | 0.39 - 3.82 |
| Moderate pain | 2.96 | 1.85 - 4.72 | 12.09 | 1.97 - 74.30 | 2.83 | 1.95 - 4.12 | 0.97 | 0.18 - 5.19 |
| Severe pain | 4.00 | 1.99 - 6.70 | na | na | 5.18 | 3.58 - 7.50 | 4.04 | 0.94 - 17.35 |
| R^2^/R^2^ adjusted | 0.029/0.029 | | 0.017/0.017 | | 0.030/0.030 | | 0.014/0.013 | |

**Supplementary Table 9.** Sub-group analyses by main language spoken at home.

**Supplementary Table 9.** Sub-group analyses by geographic location.

|  | **Self-harm in the last twelve months** | | | | **Suicidal ideation in the last twelve months** | | | |
| --- | --- | --- | --- | --- | --- | --- | --- | --- |
| **Location** | **Major cities** | | **Others** | | **Major cities** | | **Others** | |
| **Bodily pain in the last 4 weeks** | **Odds ratio** | **95% CI** | **Odds ratio** | **95% CI** | **Odds ratio** | **95% CI** | **Odds ratio** | **95% CI** |
| None (Reference) | - | - | - | - | - | - | - | - |
| Mild pain | 3.01 | 1.89 - 4.79 | 1.12 | 0.50 - 2.48 | 2.24 | 1.55 - 3.24 | 1.01 | 0.52 - 1.93 |
| Moderate pain | 4.37 | 2.62 - 7.28 | 1.50 | 0.53 - 4.27 | 3.34 | 2.25 - 4.98 | 2.02 | 0.83 - 4.89 |
| Severe pain | 5.24 | 2.64 - 10.43 | 1.78 | 0.61 - 5.15 | 6.25 | 4.13 - 9.47 | 4.08 | 2.05 - 8.12 |
| R^2^/R^2^ adjusted | 0.029/0.029 | | 0.003/0.003 | | 0.034/0.034 | | 0.028/0.027 | |
